# Sulfur Deficiency Increases Phosphate Accumulation, Uptake, and Transport in *Arabidopsis thaliana*

**DOI:** 10.3390/ijms21082971

**Published:** 2020-04-23

**Authors:** Alaa Allahham, Satomi Kanno, Liu Zhang, Akiko Maruyama-Nakashita

**Affiliations:** 1 Department of Bioscience and Biotechnology, Faculty of Agriculture, Kyushu University, 744, Motooka, Nishi-ku, Fukuoka 819-0395, Japan; allahham.alaa.706@s.kyushu-u.ac.jp (A.A.); zhang.liu.503@s.kyushu-u.ac.jp (L.Z.); 2 Institute for Advanced Research, NAIAS, Nagoya University, Frocho, Chikusa, Nagoya 464-8601, Japan; kanno.satomi@iar.nagoya-u.ac.jp

**Keywords:** *Arabidopsis thaliana*, phosphate accumulation, phosphate transporters, phosphorus, sulfur

## Abstract

Recent studies have shown various metabolic and transcriptomic interactions between sulfur (S) and phosphorus (P) in plants. However, most studies have focused on the effects of phosphate (Pi) availability and P signaling pathways on S homeostasis, whereas the effects of S availability on P homeostasis remain largely unknown. In this study, we investigated the interactions between S and P from the perspective of S availability. We investigated the effects of S availability on Pi uptake, transport, and accumulation in *Arabidopsis thaliana* grown under sulfur sufficiency (+S) and deficiency (−S). Total P in shoots was significantly increased under −S owing to higher Pi accumulation. This accumulation was facilitated by increased Pi uptake under −S. In addition, −S increased root-to-shoot Pi transport, which was indicated by the increased Pi levels in xylem sap under −S. The −S-increased Pi level in the xylem sap was diminished in the disruption lines of *PHT1;9* and *PHO1*, which are involved in root-to-shoot Pi transport. Our findings indicate a new aspect of the interaction between S and P by listing the increased Pi accumulation as part of −S responses and by highlighting the effects of −S on Pi uptake, transport, and homeostasis.

## 1. Introduction

Phosphorus (P) and sulfur (S) are essential macro elements required for plant growth and development. These elements are involved in many essential biochemical processes, and their absence severely affects plant growth and development [1,2]. Plants acquire P in the form of phosphate (Pi) which is involved in the production of nucleic acids, phospholipids, and energy providing molecule ATP [3,4,5]. In the case of S, sulfate is the major form acquired by plants [6,7]. Unlike animals, plants can reduce sulfate and produce the S-containing amino acids, such as cysteine and methionine, which are required for protein synthesis [6,7]. Thereby, reduced S in plants constitutes the main source of S in the animal’s diet [8]. Reduced S is involved in many organic compounds in plants ranging from cofactors to plant defensive compounds [6,7,9,10,11]. In addition, glutathione, a primary S metabolite, plays important roles for the detoxification of reactive oxygen species and heavy metals [11]. Both S and P play essential roles in photosynthesis, protein modification, and cellular signaling [1,2,3,5,7,12].

Considering their essential roles, plants have developed a series of complex and tightly controlled mechanisms, to meet the plant requirement of S and P in response to their availability. Recent studies have shown the effects of P deficiency (−P) on S metabolism and signaling, i.e., the increase in S and the transcript levels of several sulfate transporters by −P [13,14]; the involvement of PHOSPHATE STARAVATION RESPONSE 1 (PHR1), a key transcriptional factor for −P response, in the stimulation of interorgan sulfate transport under −P [14]; and the rapid replacement of phospholipids by galactolipids and sulfolipids under −P [15,16,17,18]. In contrast, S deficiency (−S) does not induce the replacement of sulfolipids by phospholipids in *Arabidopsis* [10]. The transcript levels of several –P-induced micro RNAs (miRs), *miR399*, *miR827*, and *miR2111*, which regulate −P responses, are downregulated under −S [19], vice versa, that of *miR395* regulating sulfate transport and activation via the induction by −S is downregulated by −P [20]. Such changes in S homeostasis and signaling contribute to plant adaptation to −P. For example, the replacement of phospholipids by sulfolipids support the cell membrane under −P. Also, excessive accumulation of metals and reactive oxygen species occurs under −P, triggers the need for more antioxidants to cope with these hazardous substances [21,22]. S is essential for the production of phytochelatin and glutathione, a major antioxidant in plants [11]. The latter is known to increase under −P, contributing to plants’ adaptation to −P [23]. While studying the interactions between S and P have mostly focused on the influence of P availability on S metabolism and homeostasis [14,15,16,17,19,20], −S is also expected to affect the P metabolism and homeostasis. However, to this date, the effects of S availability on P metabolism and homeostasis have not yet been elucidated.

Several high- and low-affinity Pi transporters (PHTs) are required for Pi uptake and transport in plants [24,25,26]. In *Arabidopsis*, nine genes of the *PHT1* family encode high-affinity plasma membrane Pi transporters [5]. Among these, PHT1;1 and PHT1;4 facilitate Pi uptake under −P [27,28]. *PHT1;2* is expressed in root epidermal cells and shares 97.7% similarity with *PHT1;1* at the nucleotide level [28,29]. PHT1;3 is suggested to be involved in reabsorbance of Pi leaked from xylem in the stele [29]. PHT1;5 functions in Pi translocation from the source to the sink tissues [30]. *PHT1;6* and *PHT1;7* are highly expressed in flowers and pollen grains, thereby suggesting their roles in Pi transport to these tissues [29]. While PHT1;8 and PHT1;9 are considered to mediate Pi acquisition by roots under −P [31], a recent study indicated the role of PHT1;8 and PHT1;9 in root-to-shoot Pi transport [32]. *PHOSPHATE1* (*PHO1*) encodes another class of Pi transporters involved in Pi transport to shoots [33,34,35]. Interestingly, the inefficient Pi transport to the shoots in *pho1* is recovered when Pi concentration is more than 1000 µM in the growth media [33].

Under −S, shoot growth is strongly retarded, whereas the numbers of lateral roots and root hairs are slightly increased without affecting the primary root growth [36,37]. Many metabolic processes are affected under −S, e.g., photosynthesis rate decreased and photorespiration is increased [7,10]. Cysteine and glutathione levels have been shown to decrease under −S [6,7,11,38]. To cope with −S, sulfate uptake and assimilation are stimulated along with the release of sulfate stored in the vacuole to the cytosol, and also the recycling of S is stimulated by degrading the secondary S metabolites such as glucosinolates, and by repressing glucosinolates synthesis [1,10,11,38,39,40,41,42,43]. These changes in sulfate transport, assimilation, and metabolism under −S is regulated by an ethylene-insensitive 3-like family transcription factor, SULFUR LIMITATION1 (SLIM1) [44].

In this study, we analyzed the effects of S availability on Pi accumulation, uptake, and transport to shoots. We found increased Pi accumulation in shoots accompanied by increased Pi uptake and Pi levels in the xylem sap under −S. Two Pi transporters involved in this alteration of Pi distribution were identified. Our study demonstrates a new aspect of the crosstalk between S and P by listing Pi accumulation as a part of plant response to −S.

## 2. Results

### 2.1. Sulfur Deficiency Increased Pi Accumulation in Shoots

**Figure 1:**
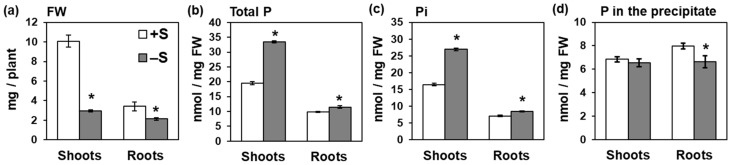
Sulfur deficiency increased phosphate (Pi) accumulation in *Arabidopsis*. (**a**) Fresh weights (FW), (**b**) total phosphorus (P) concentration, (**c**) Pi concentration, and (**d**) P concentration in the precipitate in shoots and roots of wild-type plants. Plants were grown for 10 days on MGRL agar media supplemented with 1500 μM (sulfur sufficiency (+S), white bar) or 15 μM sulfate (sulfur deficiency (−S), gray bar). In (**b**), plant samples were directly digested with concentrated nitric acid. In (**c**) and (**d**), plant tissues were extracted using 10 mM HCl. Then after transferring the supernatant to a new tube, the precipitate was digested with concentrated nitric acid. Digested plants (**b**), supernatant (**c**), and digested precipitate (**d**) were analyzed by ion chromatography. Bars and error bars indicate mean ± SE (*n* = 4). Asterisks indicate significant differences between +S and –S detected by Student’s *t*-test (* *p* < 0.05).

To understand the effects of S availability on P accumulation in *Arabidopsis*, we analyzed total P and Pi levels in shoots and roots under different S conditions. Total P and Pi analyses were performed separately. Shoot fresh weights substantially decreased under −S, while root fresh weights were either slightly decreased or not changed (Figure 1a). Total P level increased in both shoots and roots under –S, with a greater change in shoots than in roots (Figure 1b).

Total P accumulation in shoots reproducibly increased by –S, while that in roots did not (data not shown). Similar to total P, Pi level increased in shoots and roots under –S, with a higher increase in shoots (Figure 1c). In contrast, P level in the insoluble fraction was not affected by –S in shoots, and it slightly decreased in roots under –S (Figure 1d). Unlike Pi, nitrate level was not affected by –S, but sulfate level considerably decreased under –S (Figure S1). These results highlighted Pi accumulation in shoots as part of plant response to –S.

### 2.2. Enhancement of Pi Uptake under –S

**Figure 2:**
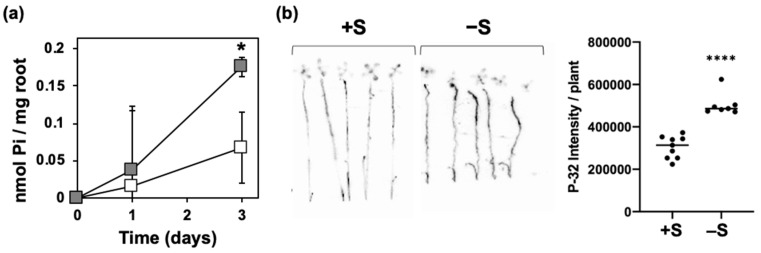
Increased Pi uptake under –S. Wild-type (WT) plants were used. In (**a**), fifteen-day-old plants were transferred to hydroponic media supplemented with 1500 μM sulfate (+S) for 2 days before starting the treatment. For the treatment, plants were transferred to hydroponic media with (+S, white color) or without (–S, gray color) sulfate. Pi uptake was calculated as described in the Materials and Methods section. In (**b**), plants were grown for 10 days on MGRL agar media supplemented with 1500 μM (+S) or 15 μM sulfate (–S). Plants were then transferred to +S or –S hydroponic medium containing ^32^P for 60 min. Bars and error bars indicate mean ± SE (for (**a**): *n* = 3, for (**b**): *n* = 7–8). Asterisks indicate significant differences between +S and –S detected by Student’s *t*-test (* *p* < 0.05) for (**a**) and Welch’s *t*-test (**** *p* < 0.0001) for (**b**).

The increased levels of total P and Pi in shoots suggested a higher Pi acquisition under –S (Figure 1). To support this hypothesis, we analyzed Pi withdrawal from the hydroponic media under +S and –S (Figure 2a). Fifteen-day-old plants were transferred to +S hydroponic media 2 days before replacement with +S or –S hydroponic media. Pi levels in the media were analyzed at 0, 1, and 3 days after starting the treatment. The decrease of Pi levels in the media from the starting day (0 day) was calculated as the amount of Pi absorbed by the roots. Pi acquisition increased about three-fold under –S compared with that under +S (Figure 2a). 

Furthermore, we analyzed the Pi uptake using the radioactive isotope of phosphorus (^32^P) ([45,46], Figure 2b). Plants grown for 10 days on +S or –S agar media were transferred to +S or –S hydroponic solution containing ^32^P. After 60 min, the intensity of ^32^P were significantly higher in plants subjected to –S comparing to that subjected to +S (Figure 2b), indicating an enhancement of Pi uptake as an early response to –S.

### 2.3. Increased Root-to-Shoot Pi Transport under –S

**Figure 3:**
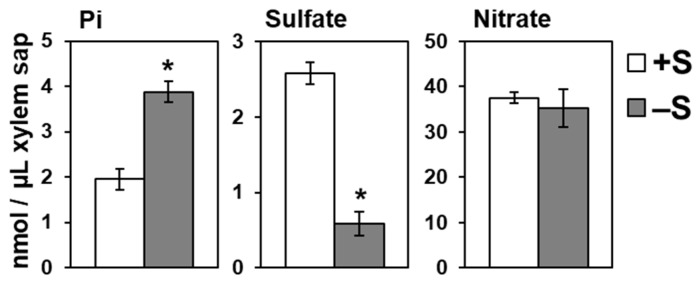
Increased Pi levels in xylem sap under –S. Wild-type (WT) plants were used. Twenty-day-old plants were transferred to hydroponic media supplemented with 1500 μM sulfate (+S) for 2 days before starting the treatment. For the treatment, plants were transferred to hydroponic media with (+S, white bar) or without (–S, gray bar) sulfate. Xylem sap was collected from decapitated stem after cutting the main stem when the main stem height reached approximately 3 cm for all plants. Bars and error bars indicate mean ± SE (*n* = 3). Asterisks indicate significant differences between +S and –S detected by Student’s *t*-test (* *p* < 0.05).

Despite the enhanced Pi uptake under –S, Pi accumulation was remarkably higher in shoots comparing to that in roots, suggesting an enhancement in root-to-shoot Pi transport under –S (Figure 1). Thus, we analyzed Pi level in xylem sap of plants grown under different S conditions (Figure 3). Twenty-day-old plants were transferred to +S or –S hydroponic media. Xylem sap was collected from decapitated stems after cutting the main stem when it became approximately 3 cm in height. Pi level in xylem sap was significantly increased in WT under –S (Figure 3), indicating that the root-to-shoot Pi transport was stimulated under –S. In contrast, sulfate level was strongly decreased in xylem sap and nitrate level was not influenced by –S (Figure 3). 

### 2.4. PHT1;9 and PHO1 Were Involved in the –S-Increased Pi Accumulation in Xylem Sap

We further investigated the effects of –S on the transcript levels of *PHT1* family members, which are involved in Pi uptake and root-to-shoot transport, and *PHO1* in roots of plants grown under S sufficiency (+S) and –S (Figure S2). Although Pi uptake significantly increased under –S, the transcript levels of the main Pi uptake transporters (*PHT1;1, PHT1;2*, and *PHT1;4*) were not affected under –S (Figure S2). Furthermore, the higher accumulation of Pi in shoots compared to that in roots, and the increased Pi levels in xylem sap, suggested an increase in Pi transport from root to shoot (Figure 1 and Figure 3). However, the transcript levels of *PHT1;8*, *PHT1;9*, and *PHO1*, which are involved in Pi transport from root to shoot, were not affected by –S (Figure S2). No signals were detected for *PHT1;8* in roots at this stage (Figure S2). The efficiency of –S was confirmed based on the transcript levels of several –S responsive genes, namely, *BGLU28*, *SDI1*, and *SULTR1;1* ([39,43], Figure S2).

To clarify the contribution of PHT1s to the increased Pi uptake under –S, we analyzed total P and Pi levels separately in T-DNA insertion mutants lacking PHT1;1, PHT1;2, and PHT1;4 grown under +S or –S (Figure S3). Total P and Pi levels in shoots increased in all mutants under –S as in wild-type (WT) plants (Figure S3) as well as a similar decrease of fresh weights and growth phenotypes (Figures S3 and S4), thereby suggesting that the disruption of a single PHT was not enough to stop the –S-increased Pi uptake. Total S decreased in shoots but not in roots (Figure S5).

**Figure 4:**
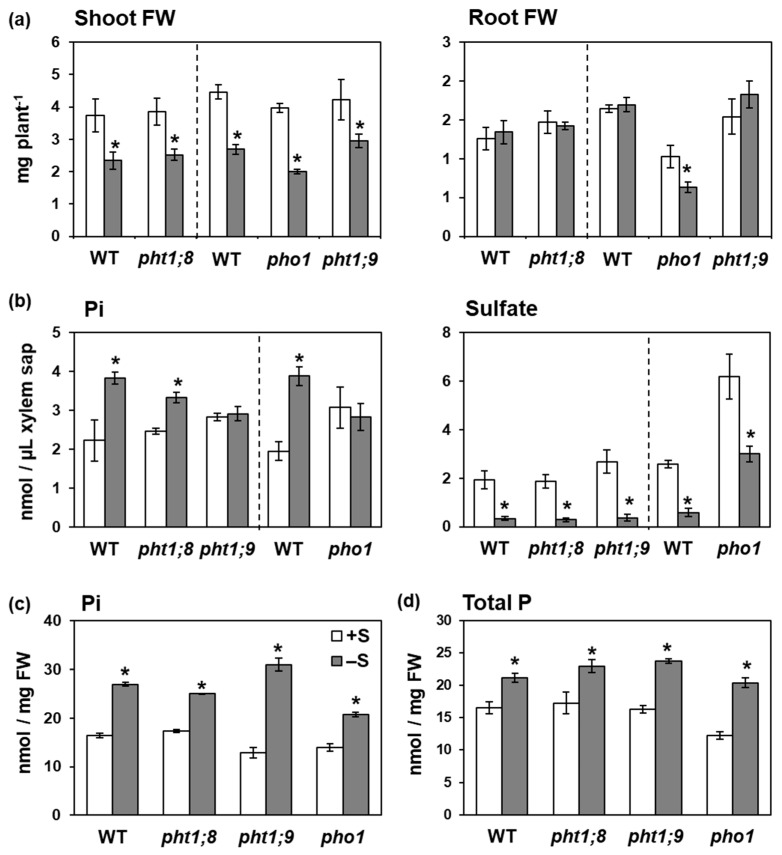
Effects of –S on Pi levels in xylem sap and shoots of wild-type (WT) plants and T-DNA insertion mutants of *PHT1;8*, *PHT1;9*, and *PHO1*. (**a**) Fresh weight (FW) of shoots (**left**) and roots (**right**). (**b**) Pi levels in xylem sap were analyzed as described in Figure 3. (**c**) Pi and (**d**) total P concentrations in shoots were analyzed as described in Figure 1. Bars and error bars indicate mean ± SE (*n* = 3). Dashed lines indicate separate experiments. Asterisks indicate significant differences between +S and –S detected by Student’s *t*-test (* *p* < 0.05).

To clarify the possible involvement of PHT1;8, PHT1;9, and PHO1 to the increased root-to-shoot Pi transport under –S, we further analyzed Pi levels in xylem sap using T-DNA insertion mutants of *PHT1;8*, *PHT1;9*, and *PHO1* (Figure 4). Fresh weights and growth phenotypes of all mutants were similar to those of WT, except of *pho1* which had abnormal shoots with short roots (Figure 4a and Figure S4, [33]). Interestingly, the –S-increased Pi level in xylem sap vanished in *pho1* and *pht1;9*, while it was detected in *pht1;8* and WT plants (Figure 4b). Sulfate levels were decreased and nitrate levels were not influenced by −S in xylem sap of all plant lines (Figure 4b and Figure S6). These results indicated the involvement of PHT1;9 and PHO1 in increasing Pi levels in xylem sap under −S.

To see how the −S-increased root-to-shoot Pi transport affect the −S-increased Pi accumulation in shoots, we further analyzed total P and Pi levels separately in *pht1;8*, *pht1;9*, and *pho1* mutants (Figure 4c,d). All mutants showed increased total P and Pi levels in shoots under −S similar to those in WT plants, despite the interruption of −S-increased root-to-shoot Pi transport in *pht1;9* and *pho1* (Figure 4b). Total S decreased under −S in shoots of all genotypes (Figure S5). These results suggested the existence of additional mechanisms underlying the Pi accumulation in shoots under −S other than the increased root-to-shoot Pi transport via PHT1;9 and PHO1.

### 2.5. Increase in Pi Accumulation by –S Was Reversed by Sulfate Addition

**Figure 5:**
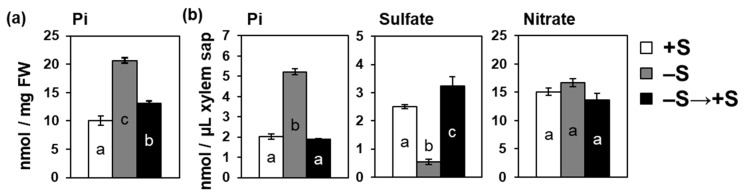
Resupplying of sulfate reversed the effects of –S on Pi accumulation in shoots (**a**) and xylem sap (**b**). Twenty-day-old plants were transferred to hydroponic media supplemented with 1500 μM sulfate (+S) for 3 days before starting the treatment. For treatments, plants were divided into three sets; +S (white bar), kept under +S for additional 3 days; –S (gray bar), transferred to hydroponic media without sulfate (–S) and kept for 3 days and –S→+S (black bar), transferred to –S media, kept for 2 days, and transferred to +S media for additional 1 day. All plants were harvested on the same day. Bars and error bars indicate mean ± SE (*n* = 4). Different letters indicate significant difference detected by the Tukey–Kramer test (* *p* < 0.05).

To confirm that the increased Pi accumulation in shoots and xylem sap was induced by –S, we investigated the effects of sulfate addition on Pi accumulation in plants subjected to –S (Figure 5). Twenty-day-old plants were transferred to –S or +S hydroponic media for 3 days before harvesting. A set from the plants transferred to –S hydroponic media was then transferred to +S media 2 days after the first transfer and kept there for 1 day (–S→+S). The increased Pi level by –S was terminated by the addition of sulfate in both shoots and xylem sap (Figure 5). Sulfate levels in the xylem sap decreased under –S and then increased again by the sulfate supply (Figure 5b). Nitrate levels in xylem sap were not influenced by S availability (Figure 5b). These results indicated that the Pi levels in shoots and xylem sap were influenced by S availability.

### 2.6. Increased Pi Accumulation under −S Was Observed in SLIM1 Mutants

**Figure 6:**
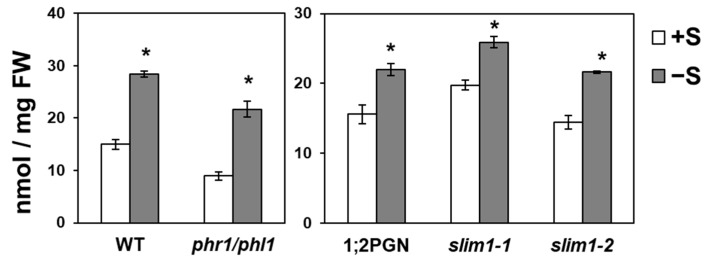
Effects of *SLIM1* and *PHR1/PHL1* disruption on –S-increased Pi accumulation in shoots. Plant growth and Pi analysis were performed as described in Figure 1. 1;2PGN represents *P_SULTR1;2_-GFP*, the parental line of *slim1* mutants [44,49]. Bars and error bars indicate mean ± SE (*n* = 4). Asterisks indicate significant differences between +S and –S detected using Student’s *t*-test (* *p* < 0.05).

PHR1/PHL1 and SLIM1 are the main transcription factors regulating −P and −S responses, respectively [44,47,48]. Therefore, we further evaluated the possible involvement of PHR1/PHL1 or SLIM1 in regulating the −S-increased Pi accumulation in shoots using *slim1-1*, *slim1-2*, *slim1* parental line *P_SULTR1;2_-GFP* (1;2PGN), and *phr1/phl1* ([44,47,48,49], Figure 6). Pi levels in shoots of *slim1* and *phr1/phl1* mutants significantly increased under −S similar to that of 1;2PGN and WT plants (Figure 6), suggesting the existence of a different mechanism regulating −S-increased Pi accumulation in shoots. 

## 3. Discussion

**Figure 7:**
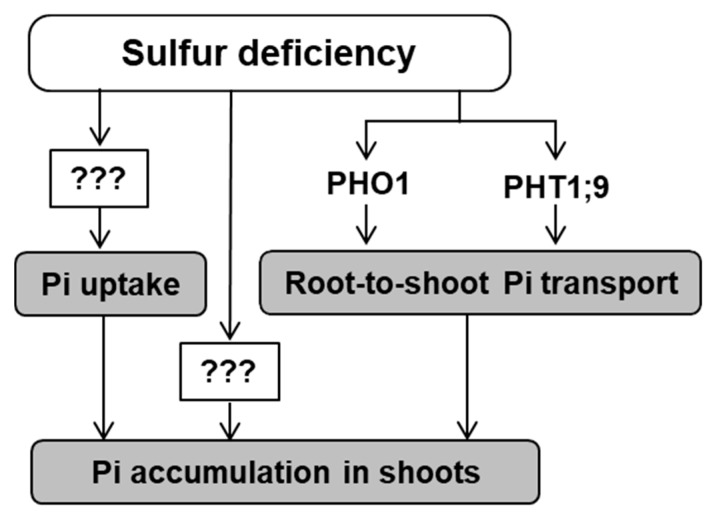
Effects of –S on Pi accumulation, uptake, and transport to shoot. Under –S, higher Pi uptake and Pi transport from roots to shoots were confirmed in this study, which ultimately leads to higher Pi accumulation in shoots under –S listing it as part of –S response in plants. Two transporters, namely, PHT1;9 and PHO1, were found to contribute to the increased Pi transport from root to shoot under –S. Further investigations required to identify the molecular mechanisms regulating the increased Pi uptake and accumulation under –S. Arrows (→) indicates “stimulated by –S”.

We investigated the effects of S availability on Pi accumulation, uptake, and transport to shoots in *A. thaliana* and demonstrated the increased Pi uptake and Pi accumulation in shoots and xylem sap under −S (Figure 1, Figure 2, and Figure 7), thereby highlighting crosstalk between S and P under −S. The higher Pi accumulation in plants growing under −S compared to that in plants growing under normal conditions can be supported by higher Pi acquisition under −S (Figure 1 and Figure 2). Besides, the −S-increased Pi accumulation was still observed in the *PHT1;1*, *PHT1;2*, and *PHT1;4* single mutants (Figure S3), suggesting the involvement of several Pi transporters in the increased Pi uptake or another class of Pi transporters. Also, the transcript levels of *PHT1;1, PHT1;2*, and *PHT1;4*, the high affinity Pi transporters facilitate Pi uptake from roots, were not affected under −S (Figure S2). Several posttranslational regulators are capable of physically interact with PHT1 proteins [50,51,52,53,54,55]. *PHT1* members are capable of physically interact in the plasma membrane and form homomeric and heteromeric complexes, in dicot plants, providing an additional posttranslational regulatory mechanism for these transporters [25,56,57,58,59]. In addition, Pi uptake might be stimulated under −S by the increased number of lateral roots and root hairs [36,37], as Pi uptake was reported to be improved by the enhanced lateral root and root hair formation under –P [36,60,61].

The −S-increased Pi accumulation was remarkably higher in shoots compared with that in roots (Figure 1b). The increased Pi level in xylem sap indicated an increase in root-to-shoot Pi transport under −S, which probably led to increased Pi accumulation in shoots. Although PHO1 is involved in Pi transport to shoots, the Pi level in xylem sap of *pho1* was similar to that in xylem sap of WT (Figure 4b). This result is consistent with that reported in a previous study, which showed normal Pi transport to shoots in *pho1* when Pi concentration was more than 1000 µM in the growth media [33]. The −S-increased Pi level in the xylem sap disappeared in *pht1;9* and *pho1* (Figure 4b), indicating their involvement in this increase (Figure 7). Although PHO1 and PHT1;9 proteins were functional in *pht1;9* and *pho1*, respectively, disruption of either of them was sufficient to interrupt the –S-increased Pi accumulation in xylem sap (Figure 4b). These results suggest that PHO1 and PHT1;9 possibly work together, either as complex or by regulating one another, to regulate the increased Pi accumulation in xylem sap under −S. Previous studies suggested that PHO1 is involved in the long distance signaling cascade and might have additional biological or regulatory role beside the root-to-shoot Pi transporting properties [62,63,64,65]. This could further explain the stability of the severe phenotype observed in *pho1* despite the normal Pi transport to shoots in *pho1* under Pi sufficiency [33]. In addition to PHO1, disruption of *PHT1;9* has been reported to affect the transcript profile of several −P responsive genes, indicating a possible regulatory role of PHT1;9 [32]. Intriguingly, despite the interruption of the −S-increased Pi level in the xylem sap, total P and Pi accumulation in shoots were not affected in *pht1;9* and *pho1* (Figure 4c,d). This result suggests an additional mechanism underlying the increased Pi accumulation in shoots other than the increased Pi uptake and Pi transport to shoots, for example, the interruption in shoot-to-root Pi transport (Figure 7). 

The −S-increased Pi uptake, Pi transport to shoots, and Pi accumulation in shoots raise the question as to why plants accumulate more Pi under −S. Both P and S play essential roles in plant growth and development by being involved in the production of essential compounds, such as amino acids and lipids, and key metabolic processes, such as photosynthesis and photorespiration [1,2,3,5,7,12]. Given the vital role of these essential nutrients, the interactions and coordination between S and P in response to their deficiencies could be essentially required to sustain a better plant growth and development under these conditions. For example, supporting cell membrane via replacing phospholipids by sulfolipids under −P [15,16,17,18]; and alleviating the effects of over-accumulated metals and reactive oxygen species under −P through the accumulation of glutathione [23]. The increased S accumulation in roots has been reported under −P [14], whereas our study indicated increased Pi accumulation in shoots under −S (Figure 1). Pi accumulation might be required to sustain plant growth under −S, or it can be an indirect consequence of plant response to −S. At this point, it is difficult to propose the real physiological meaning of the increased Pi accumulation in shoots under −S. Confirming that the increased Pi in shoots and xylem sap was a response to −S, the re-addition of sulfate to plants subjected to −S recovered the Pi levels in shoots and xylem sap to that observed under +S (Figure 5). The increased sulfate levels in *pho1* also indicated the negative interaction between sulfate and Pi (Figure 4b). However, this phenomenon was not affected in *slim1* or *phr1/phl1* (Figure 6), indicating the existence of another pathway independent of SLIM1-regulated plant response to −S and PHR1/PHL1. Considering the close ionic status between sulfate and Pi (SO_4_^2−^ and PO_4_^3−^), another interesting hypothesis is the requirement of Pi accumulation to maintain the cellular or subcellular ionic balance under −S, vice versa occurs under −P. 

## 4. Materials and Methods 

### 4.1. Plant Materials and Growth Conditions

*Arabidopsis thaliana*, cv. Columbia was used as wild-type (WT) plants. The seeds of T-DNA insertion mutants, *pht1;1* (SALK_088586C), *pht1;2* (SALK_110194C), *pht1;4* (SALK_103881C), *pht1;8* (SALK_056529C), *pht1;9* (SALK_073614C), and *pho1* (SALK_080534C) were obtained from the Arabidopsis Biological Resource Center (ABRC). *slim1-1*, *slim1-2*, and their parental line *P_SULTR1;2_-GFP* (1;2PGN) were isolated previously [44,49]. The seeds for *phr1/phl1* were kindly provided by Dr. Javier Paz-Ares (National Center of Biotechnology, Spain) [47,48].

For the analysis of total P, Pi, and transcript levels, seeds were sterilized and sown on MGRL media [66,67] containing 1% sucrose and 0.8% agar. For media preparation, the agar was washed and vacuum filtrated using 5 L deionized and 1 L distilled water to remove the sulfate in the agar. Supplemented sulfate concentration was adjusted to 1500 μM (S sufficient, +S) or 15 μM (S deficient, –S) in the form of MgSO_4_. In –S medium, Mg^2+^ was supplied as MgCl_2_ up to 1500 μM. Plants were vertically grown for 10 days at 22 °C with constant light (40 µmol m^−2^ s^−1^). 

For preparing the hydroponic media used in Pi uptake assay (Figure 2a) and xylem sap analysis (Figure 3 and Figure 4b), the MGRL media with the same mineral nutrient composition excluding agar and sucrose was used. Plants were allowed to grow for at least 2 days on full nutrition hydroponic media before starting the treatments. For the treatments, plants were transferred to hydroponic media supplemented with 1500 μM (+S) or 0 μM (–S) sulfate. 

### 4.2. Analysis of Total P, Pi, Sulfate, and Nitrate in Plant Tissues

Shoots and roots were separated and divided into 4 replicates, in average 15 plants per replicate. The weight for each replicate was recorded before frozen with liquid nitrogen. 

For total P analysis, plant tissues were directly digested after harvesting with 200 µL concentrated nitric acid (Nacalai Tesque, Kyoto, Japan) at 95 °C for 30 min followed by the evaporation at 115 °C till approximately 10 µL was remained in the tube. The digested samples were then diluted to 1 mL using ultra-pure water. 

For Pi, sulfate, and nitrate analysis, frozen tissues were mechanically ground to fine powder using Tissue Lyser (Retsch, Germany). Ground tissues were extracted at 4 °C with 5 times the volume of 10 mM HCl to the fresh weight. Supernatant was separated from cell debris (precipitate) by centrifuging at 4 °C, 12,000 rpm, for 10 min. After complete separation, cell debris was digested with 200 µL concentrated nitric acid as described for total P analysis.

Pi, sulfate, and nitrate were analyzed using ion chromatography (IC-2001, TOSOH, Yamaguchi, Japan) as previously described [68]. For the analysis of supernatant, anions were separated at 40 °C using a TSK SuperIC-AZ column (TOSOH), flow rate at 0.8 mL min^−1^, serial 30 µL injections, with the eluent containing 1.9 mM NaHCO_3_ (Wako Pure Chemicals, Osaka, Japan) and 3.2 mM Na_2_CO_3_ (Wako Pure Chemicals). For the analysis of digested samples, the eluent containing 7.5 mM NaHCO_3_ and 1.1 mM Na_2_CO_3_ was used. Anion mixture standard solution 1 (Wako Pure Chemicals) was used as a standard. 

Pi concertation in the T-DNA insertion mutants was measured using ascorbic acid methods in Figure 4, Figure 6 and Figure S3 [69]. Using a 1.5 mL tube, 20–200 µL supernatant were mixed up to 840 µL with distilled water, then 160 µL chromogenic mixture was added. After 15 min, the absorbance was measured at 710 nm using SpectraMax 340PC Microplate Reader (Molecular Devices, San Jose, CA, USA). For the measurement, 200 µL of the prepared mixture was loaded into a 96-well plate (Iwaki, Shizuoka, Japan). The chromogenic mixture was prepared as previously described [69].

### 4.3. Analysis of Transcript Level

Plants were grown for 10 days on +S or –S MGRL media. Shoots and roots were harvested separately and divided into 3 replicates, in average 15 plants per replicate. Frozen tissues were mechanically ground to fine powder using Tissue Lyser (Retsch). Total RNA was extracted using Sepasol-RNA I (Nacalai Tesque) followed by the reverse transcription using PrimeScript RT Reagent Kit with gDNA Eraser (Takara). For the reverse transcription, 0.25 µg of the total RNA was used for roots samples. Transcript levels were determined by quantitative real time PCR using KAPA SYBR FAST qPCR Master Mix (2×) kit (Kapa Biosystems, Cape Town, South Africa), and qTOWER^3^ real-time PCR thermal cyclers (Analytik Jena, Thuringia, Germany) using specific primers (Table S1) [32,70,71]. Relative expression was calculated by ΔΔCt method with *UBQ2* as an internal control. Blank samples were prepared with sterilized distilled water instead of samples. The representative –S responsive genes, *BGLU28*, *SDI1*, and *SULTR1;1*, were analyzed for their transcript levels as a positive control (Figure S2) [39,43].

### 4.4. Pi Uptake Analysis

Plants of similar size grown for 15 days on GM media were transferred to 6 containers containing 200 mL of +S hydroponic media, with 6 plants per container (230 mL), and these plants continued to grow for at least 2 days before starting the treatment. For treatments, the hydroponic media were replaced with 200 mL of +S or –S hydroponic media, with 3 containers for each treatment. The hydroponic containers were then placed inside of a large transparent container (33 × 15 × 10 cm) and covered with a plastic wrap to minimize evaporation loss. Plants were grown for an additional 3 days at 22 °C with constant light (40 µmol m^−2^ s^−1^). After replacing the media, 70 µL of the hydroponic media was collected from each hydroponic container at 0, 1, and 3 days. Fresh weights of plant roots in each container were recorded on the third day.

The Pi concentration in the collected medium was analyzed using ion chromatography (IC-2001, TOSOH), as described in 4.2. The Pi withdrawn from the media was calculated based on the difference between the Pi concentration at 1 or 3 days after replacing the media and the Pi concentration at 0 day from the same container. The obtained value was then divided by root fresh weight. The Pi uptake rate is expressed as the concentration of Pi withdrawn from the media by 1 mg root.

Pi uptake analysis using ^32^P was performed as described previously [45,46]. Surface-sterilized seeds were plated on +S or –S agar media and grown for 10 days. The seedlings were transferred to +S or –S MGRL hydroponic solution containing 10 kBq ml^−1 32^P-labelled orthophosphate (PerkinElmer) and incubated for 60 min under light conditions. Images of ^32^P uptake by seedlings were obtained using an imaging plate (GE Healthcare Japan, Tokyo, Japan) and a FLA-8000 fluorescent image analyzer (GE Healthcare Japan). The intensities of the ^32^P signals were quantified using ImageQuant TL (GE Healthcare Japan).

### 4.5. Xylem Sap Analysis

Plants were grown for 20 days on GM media [72], transferred to +S hydroponic media, and grown for at least 2 days. Thereafter, the plants were transferred to +S or –S hydroponic media and grown there till the main stem height reached approximately 3 cm.

Xylem sap was collected as described previously [73]. Leaves were cut with sharp scissors followed by cutting the main stem. Plants were immediately placed in a transparent container to maintain high humidity. Xylem sap droplets were directly collected from the cut stems by using a pipette. Two microliter of the collected xylem sap was diluted to 400 µL and analyzed by ion chromatography (IC-2001) as described in 4.2.

### 4.6. Statistical Analysis

The data were statistically analyzed using Excel Analysis ToolPak Add-ins in Office365 ProPlus (Microsoft). Significant difference between +S and –S was defined with *p*-values less than 0.05 detected with Student’s *t*-test ( Figure 1, Figure 2a, Figure 3, Figure 4, and Figure 6) and Welch’s *t*-test (Figure 2b). Single factor ANOVA was used to detect significant difference between +S, –S, and +S→–S in the recovery treatment (Figure 5) followed by Tukey–Kramer test at *p*-values less than 0.05.

## 5. Conclusions

This study added to the existing knowledge a new aspect of the interaction between S and P, providing the first insight on the effects of −S on Pi uptake, transport, and accumulation. We identified the −S-increased Pi accumulation in shoots as a part of −S responses in plants. This accumulation was facilitated by increased Pi uptake and root-to-shoot transport under −S. The −S-increased Pi transport from root-to-shoot was regulated by PHO1 and PHT1;9. However, our results suggested a separate mechanism underlying the Pi accumulation in shoots. Studying the interaction points between S and P can provide a better understanding of these essential nutrients and highlights the importance of such interactions in plant adaptation to their deficiencies. It can provide a powerful tool for future improvement of plants growth and development. However, further investigations are still required to confirm the physiological meaning and to identify the molecular mechanism underlying the −S-increased Pi uptake and accumulation in shoots. 

## Supplementary Materials

Supplementary materials can be found at https://www.mdpi.com/1422-0067/21/8/2971/s1.

## Abbreviations

P	Phosphorus
S	Sulfur
Pi	Phosphate
+S	Sulfur sufficiency
–S	Sulfur deficiency
–P	Phosphorus deficiency
PHT	Phosphate transporter
T-DNA	transfer DNA
RNA	Ribonucleic acid
miR	Micro ribonucleic acid
qPCR	Quantitative polymerase chain reaction
IC	Ion chromatography
WT	Wild-type
n.d.	not determined
SE	Standard error
ANOVA	Analysis of variance
GM	Germination media
µL	Microliter
µM	Micro molar
mL	Milliliter
mM	Millimolar
°C	Degrees of Celsius
