# Supplementary material for: Sulfur Deficiency Increases Phosphate Accumulation, Uptake, and Transport in Arabidopsis thaliana
Source: Int J Mol Sci. 2020 Apr 23;21(8):2971. doi: 10.3390/ijms21082971 (PMC7215917; doi:10.3390/ijms21082971)
Supplement: Supplementary file 1 [file ijms-21-02971-s001.pdf]

Supplemental Table S1. List of primers used for quantitative RT-PCR.

Gene name	Locus	Direction	Sequence (5'->3')	
PHT1;1	AT5G43350	Fwd	CCTCAACTCTCCAGAGAAGTTCTTA	[70]
		Rev	TTCGGCCATTTCCTAGAGC	
PHT1;2	AT5G43370	Fwd	AGGGCAAGTCCCTCGAAGAACT	[32]
		Rev	ATCAAACAAACCACAAACAACTCCACAT	
PHT1;3	AT5G43360	Fwd	CCAAAGGCAAGTCCCTTGAAGAACT	
		Rev	CAAAGAACGTAAAACGTAAAAGTAGTACACCATT	
PHT1;4	AT2G38940	Fwd	TTGCTCCTAATTTTCCTGATGCT	
		Rev	TGTGCCGGCCGAAATCT	
PHT1;5	AT2G32830	Fwd	CGCCGATATCCCATGACAAG	
		Rev	GACCTAATGCGACGACGTTTG	
PHT1;6	AT5G43340	Fwd	ACGTTATACATCATGGCAGGAATCAAT	
		Rev	AAGCTCCTCAAGTGATTTCCCATTAGT	
PHT1;7	AT3G54700	Fwd	TGGAGGATATCCATGCTCTGTCT	
		Rev	CGCGGCTTCTGGAAAATTAG	
PHT1;8	AT1G20860	Fwd	TTACCCGAAGTAAACCGTATGAGAA	
		Rev	AATACGTCACCAAGATTCCAGCAA	
PHT1;9	AT1G76430	Fwd	TGGAGCTGCAGGGAAGTTTG	
		Rev	ATCTGGAAAACCGTCCTCTTCAT	
PHO1	AT3G23430	Fwd	TAAGGAGATGGTGGGACGAA	[72]
		Rev	TTAACCGTCTGAGTCCCTGTC	

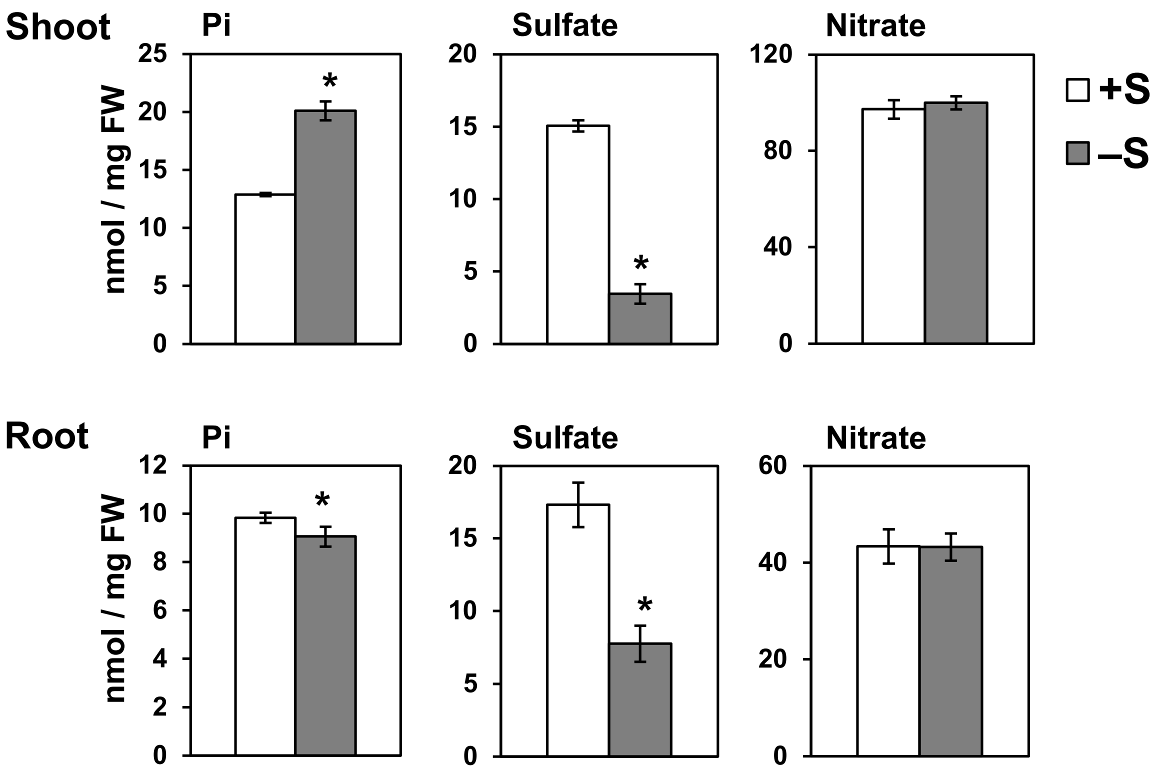

Figure S1. Concentrations of Pi, sulfate, and nitrate in shoots (upper) and roots (lower) of *Arabidopsis*. Plants were grown for 10 days on MGRL agar media supplemented with 1500 μM (+S, white bar) or 15 μM sulfate (–S, gray bar). Plants were analyzed as described in Figure 1. Bars and error bars indicate mean ± SE (n = 4). Asterisks indicate significant difference between +S and –S detected with Student's *t*-test (* $p < 0.05$).

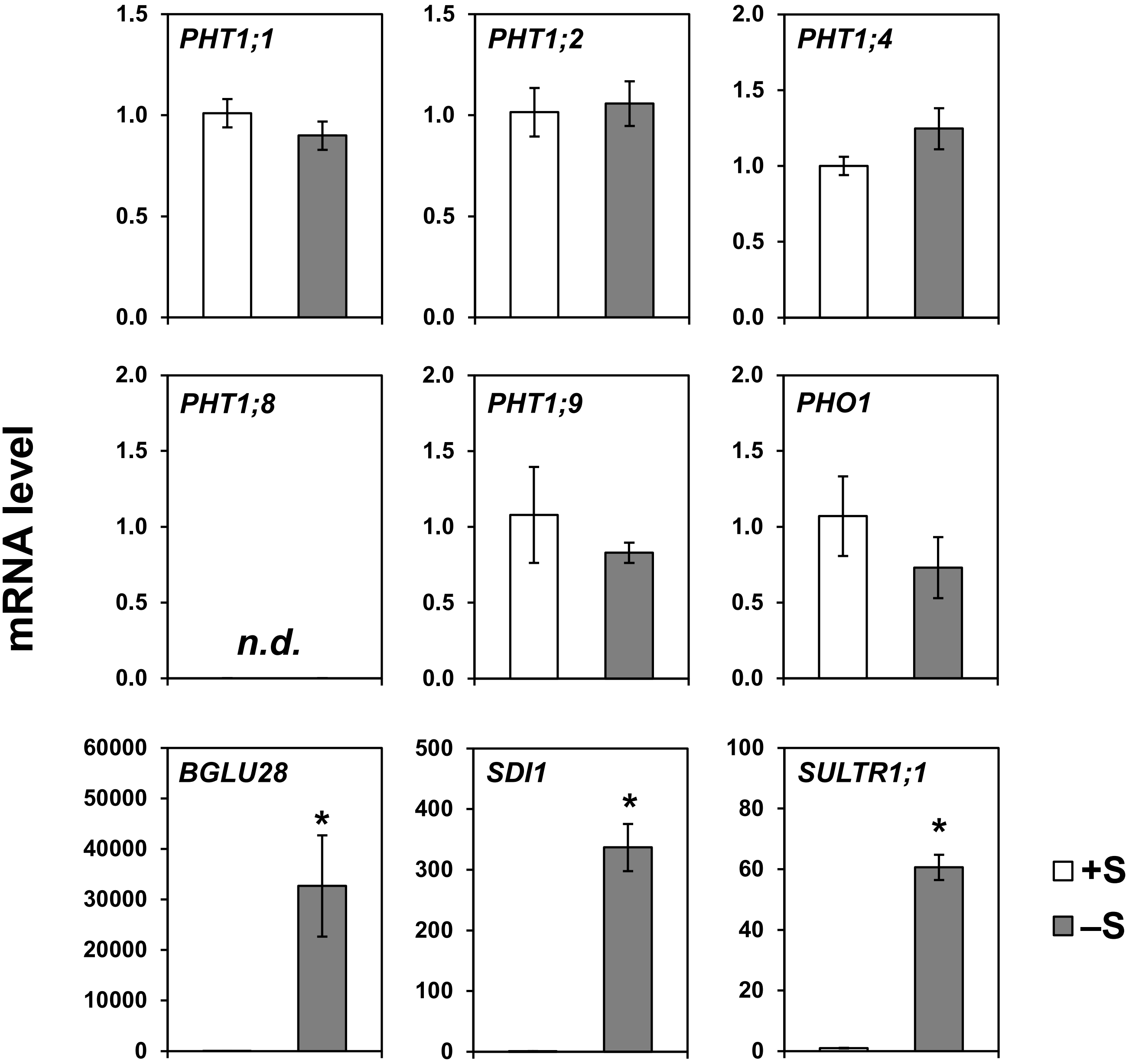

Figure S2. Effects of –S on the transcript levels of several Pi transporters in roots. Plants were grown for 10 days under +S (white bar) and –S (gray bar). Their relative expressions were analyzed by quantitative real-time RT-PCR by using *UBQ2* as the internal control. Data were analyzed by the ΔΔCt method. Bars and error bars indicate mean ± SE (n = 3), n.d. indicates "not detected". Asterisks indicate the significant differences between +S and –S detected by Student's *t*-test (* $p < 0.05$).

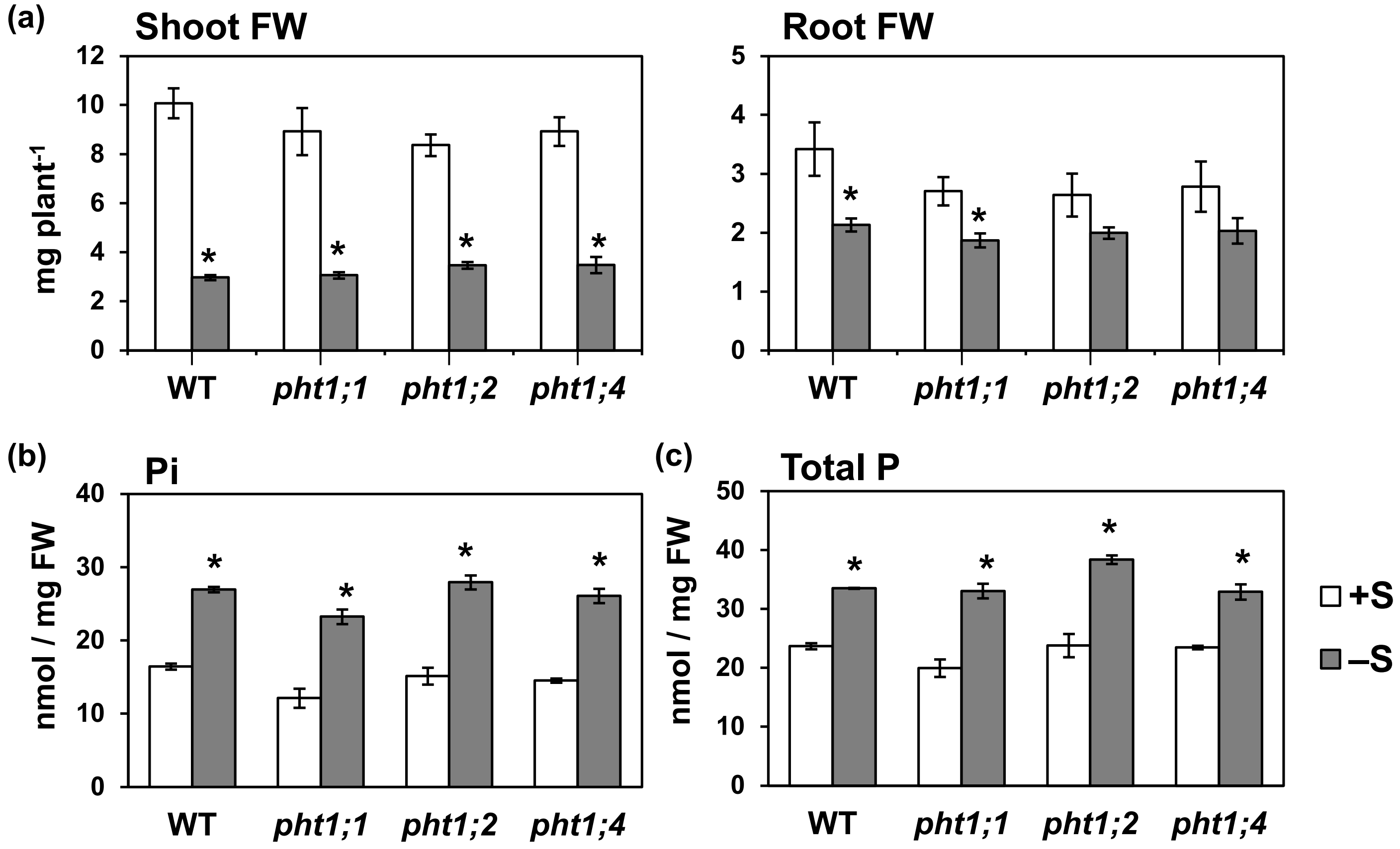

Figure S3. The effects of the disruption of Pi uptake transporters on (a) fresh weights (FW) of shoot (left) and root (right), (b) Pi, and (c) total P level in shoots. Wild-type (WT) plants and each T-DNA insertion mutant of *PHT1;1*, *PHT1;2*, and *PHT1;4*, namely, *pht1;1*, *pht1;2*, and *pht1;4*, respectively, were used. Plants were grown for 10 days on MGRL agar media supplemented with 1500 μM (+S, white bar) or 15 μM sulfate (–S, gray bar). Pi and total P were analyzed as described in Figure 1. Bars and error bars indicate mean ± SE (n = 3). Asterisks indicate significant differences between +S and –S detected by Student's *t*-test (* $p < 0.05$).

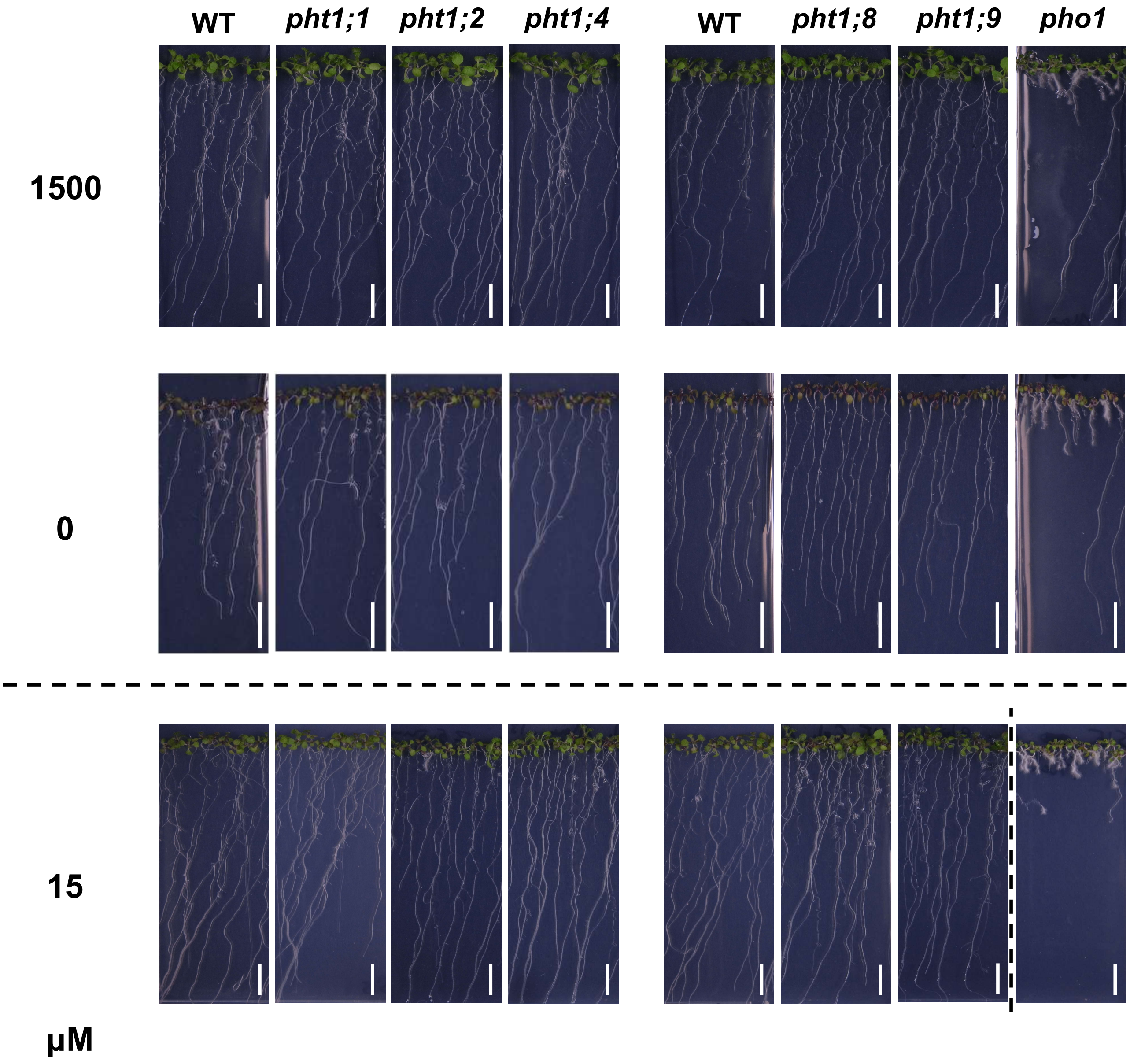

Figure S4. Plants growth phenotype under different S conditions. Plants were grown for 10 days on MGRL media supplemented with 1500 µM (upper), 15 µM (middle) and 0 µM (lower) sulfate. White lines indicate scale (1 cm).

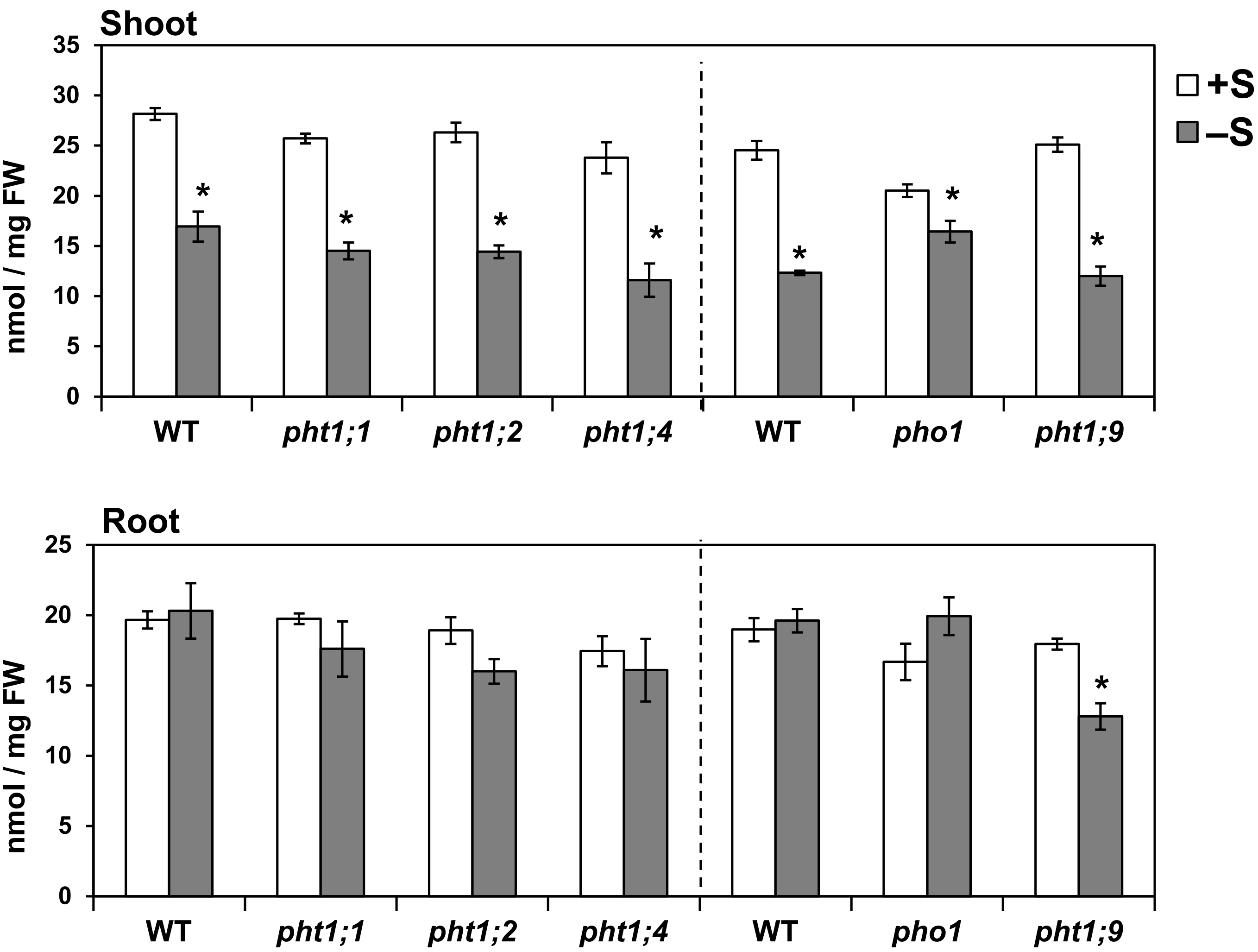

Figure S5. Total S in shoots (upper) and roots (lower) of the T-DNA insertion lines grown under +S and -S. Plants were grown for 10 days on MGRL agar media supplemented with 1500 μM (+S, white bar) or 15 μM sulfate (–S, gray bar). Bars and error bars indicate mean ± SE (n = 4). Dashed-lines indicate separate experiments. Asterisks indicate significant differences between +S and –S detected with Student's *t*-test (* $p < 0.05$).

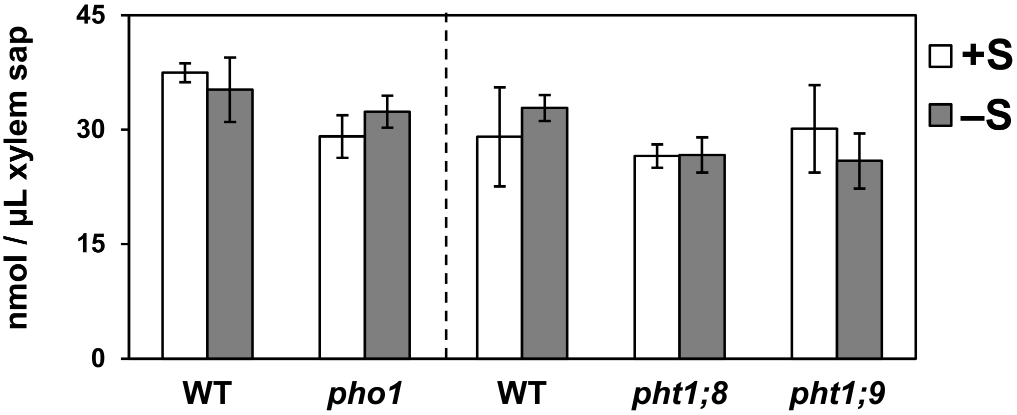

Figure S6. Nitrate concentration in xylem sap of the T-DNA insertion lines grown under +S and -S. Plants were grown as described in Figure 3. Bars and error bars indicate mean ± SE (n = 4). Dashed-lines indicate separate experiments. Asterisks indicate significant difference between +S and –S detected with Student's *t*-test (* $p < 0.05$).
